# The Presence of Vacuolated Kupffer Cells Raises a Clinical Suspicion of Niemann-Pick Disease Type C in Neonatal Cholestasis

**DOI:** 10.3389/fgene.2022.867413

**Published:** 2022-03-18

**Authors:** Neng-Li Wang, Lian Chen, Yi Lu, Xin-Bao Xie, Jing Lin, Kuerbanjiang Abuduxikuer, Jian-She Wang

**Affiliations:** ^1^ Center for Pediatric Liver Diseases, Children’s Hospital of Fudan University, Shanghai, China; ^2^ Department of Pathology, Children’s Hospital of Fudan University, Shanghai, China; ^3^ Department of Pediatrics, Icahn School of Medicine at Mount Sinai, New York, NY, United States

**Keywords:** neonatal cholestasis, infant, Niemann-Pick disease type C, Kupffer cell, diagnosis

## Abstract

Early diagnosis of Niemann-Pick disease type C (NP-C) in neonatal cholestasis is still challenging because splenomegaly is non-specific and oxysterol profiling studies also have a relatively low specificity. This study explores a method for identifying infants with a high clinical suspicion of NP-C in neonatal cholestasis. We reviewed the clinical findings of 9 neonatal cholestatic infants with NP-C genetically diagnosed between January 2015 and December 2020. Seven underwent liver biopsy at ages ranging from 35 to 112 d. Foam cells were only detected in 2 (28.6%, 2/7) liver tissues obtained beyond 3 months of age. However, vacuolated Kupffer cells were detected in all 7 liver tissues. Their significance was explored by using 168 neonatal cholestatic infants, who underwent genetic tests and liver biopsy between January 2018 and December 2020. Of them, 26 detected vacuolated Kupffer cells. Six (23.1%, 6/26) were diagnosed as NP-C, comparing to none of the 142 neonatal cholestatic infants without vacuolated Kupffer cells (*χ* ^*2*^ = 33.983, *p* < 0.001). The ratio of positive diagnosis of NP-C was 31.6% (6/19) in neonatal cholestatic infants with both vacuolated Kupffer cells and splenomegaly. Therefore, we conclude that the presence of vacuolated Kupffer cells can raise a high clinical suspicion of NP-C in neonatal cholestatic infants, especially in those with splenomegaly.

## Introduction

Niemann-Pick disease type C (NP-C) is a rare progressive and life limiting lysosomal storage disorder. It results from compound heterozygous or homozygous pathogenic variants in either of the two genes: *NPC1* or *NPC2* (Carstea et al., 1993; Vanier et al., 1996). Nearly 95% of cases are caused by NPC1 deficiency, with approximately 5% caused by NPC2 deficiency (Jahnova et al., 2014). It is classified as visceral-neurodegenerative form (early-infantile), neurodegenerative form (late-infantile and juvenile), and psychiatric-neurodegenerative form (adult) (Patterson, 2000; Geberhiwot et al., 2018). Primary manifestations are age dependent. In early infancy, clinical manifestations are predominantly visceral, with cholestasis and hepatosplenomegaly (Patterson, 2000). Cholestasis in the majority spontaneously resolves after 3–4 months of age, while splenomegaly persists and neurological symptoms develop with age (Evans et al., 2017; Geberhiwot et al., 2018). The early diagnosis is the key for reduction of organ damage since a medical treatment is available now (Pineda et al., 2018).

Newborn screening for NP-C still has not been developed. Splenomegaly in neonatal cholestatic infants raises a clinical suspicion of NP-C (Geberhiwot et al., 2018). Two plasma oxysterols, 7-ketocholesterol (7 KC) and cholestane-3β,5α,6β-triol (C-triol), are biomarkers for aiding diagnosis (Mazzacuva et al., 2016; Maekawa et al., 2020). Genetic tests can lead to a definite diagnosis. However, early diagnosing of NP-C is still challenging because splenomegaly is non-specific (Mazzacuva et al., 2016; Maekawa et al., 2020) and oxysterol profiling studies also have a relatively low specificity for NP-C in neonatal cholestasis (Polo et al., 2016; Degtyareva et al., 2019). Genetic tests are usually ordered if an inherited disorder is suspected. Therefore, an alternative is necessary for early identification of infants with a clinical suspicion of NP-C in neonatal cholestasis.

This study summarized the clinical findings of 9 NP-C infants presenting as neonatal cholestasis, and unexpectedly found that vacuolated Kupffer cells were detected in all liver tissues obtained in the early disease course. We also explored the significance of vacuolated Kupffer cells on early detection of infants with a high suspicion of NP-C in neonatal cholestasis.

## Methods

### Patients and Definitions

This study enrolled neonatal cholestatic infants (onset <3 months of age) diagnosed as NP-C who were referred to the Children’s Hospital of Fudan University between January 2015 and December 2020. The diagnosis of NP-C is established if *NPC1* or *NPC2* biallelic pathogenic/likely pathogenic variants are identified. Cholestasis is defined as follows (Togawa et al., 2016): serum direct bilirubin (DB) > 20.0% of total bilirubin (TB) if TB > 85.5 μmol/L; or DB > 17.1 μmol/L if TB < 85.5 μmol/L. Hepatomegaly and splenomegaly were diagnosed by ultrasonography.

To explore the significance of vacuolated Kupffer cells, this study also enrolled 168 consecutive infants with neonatal cholestasis, who underwent both genetic tests and liver biopsy, between January 2018 and December 2020. Following a work-up for neonatal cholestasis as described previously (Liu et al., 2010), surgical, infectious, parenteral nutrition, endocrinological, and drug-induced causes were excluded.

The study was approved by the ethics committees of the Children’s Hospital, Fudan University and conducted in full compliance with medical ethics standards. Informed consent had been obtained from the parents/guardians during the admission. Clinical data were collected from their medical records.

### Genetic Testing

Genetic testing was performed in the Translational Center of Children’s Hospital of Fudan University. Genomic DNA was extracted from peripheral blood. *NPC1* variants (NM_000271) and *NPC2* variants (NM_006432) were screened by NGS, including panel, medical exome, and whole exome sequencing. The procedures of sequencing, data analyses, and variation classification were described previously (Wang et al., 2016; Qiu et al., 2017; Zhang et al., 2020). Variant pathogenicity was assessed according to the American College of Medical Genetics and Genomics (ACMG) standards and guidelines (Richards et al., 2015).

### Histologic Studies

Liver tissues were obtained by needle biopsy or intraoperative wedge biopsy. Liver tissues sections were stained by hematoxylin and eosin (HE), periodic acid-schiff (PAS), anti-CD68 (GENE, Shanghai, China), etc.

Smears of bone marrow aspirations were Wright’s stained.

### Statistical Analysis

Statistical analysis was performed using SPSS Inc. version 17.0 software (University of Chicago, Chicago, IL). Difference among ratios was tested by Chi-square test using Fisher’s exact value. Comparison of two medians was done by nonparametric Mann-Whitney test. *p* < 0.05 was considered significant.

## Results

### Molecular Findings

A total of 9 neonatal cholestatic infants, including 4 boys and 5 girls, were finally diagnosed as NP-C for harboring biallelic pathogenic or likely pathogenic variants in *NPC1* (Table 1). No infant was found to harbor biallelic pathogenic variants in *NPC2*. Sixteen distinct *NPC1* variants were identified, including 10 known disease-causing variants and 6 novel variants (4 frameshift indels and 2 missense variants) absented from the Genome Aggregation Database (GnomAD). The 2 novel missense variants, c.1024T > C (p.W342R) and c.3254A > C (p.Y1085S), were predicted to be disease causing and damaging by MutationTaster, Polyphen-2, and SIFT. Both were rated as likely pathogenic variants, while the 4 novel frameshift indels as pathogenic variants according to the ACMG standards and guidelines.

**TABLE 1:** Molecular findings in *NPC1* (NM_000271) of 9 patients with neonatal cholestasis.

	Variant 1	Variant 2	Origin
P1	c.1757 + 3_1757+6delGAGT	**c.3254_3255delAT**	M/F
P2	**c.10delC**	c.1211G > A (p.R404Q)	ND
P3	**c.1024T > C (p.W342R)**	**c.2970_2971insTCCT**	M/F
P4	**c.3254A > C (p.Y1085S)**	**c.3254A > C (p.Y1085S)**	F/M
P5	c.1138C > T (p.L380F)	c.1211G > A (p.R404Q)	F/M
P6	c.352_353delAG	c.2000C > T (p.S667L)	ND
P7	**c.2207_2208dupTC**	c.2972_2973delAG	M/F
P8	c.1421C > T (p.P474L)	c.2728G > A (p.G910S)	ND
P9	c.1301C > T (p.P434L)	c.3425T > C (p.M1142T)	ND

P, patient; ND, not done; F, father; M, mother.

Novel pathogenic or likely pathogenic variants are shown in bold font.

### Clinical Findings

The 9 NP-C infants came from 9 distinct nonconsanguineous families. Jaundice and hepatosplenomegaly were identified in all 9 patients (Table 2). Six exhibited acholic stools. Seven, but not patient (P) 3 and P7, were classified into cholestasis with high serum γ-glutamyl transpeptidase (GGT >100U/L). Aspartate aminotransferase (AST) was elevated in all 9 patients, and the ratios of AST to alanine aminotransferase (ALT) ranged from 2.1 to 7.5. Hypoglycemia (blood glucose levels<3.0 mmol/L) was present in 4 patients (P5, P6, P8, and P9) after fasting for 3 h.

**TABLE 2:** Clinical findings of the 9 NPC patients presenting as neonatal cholestasis.

	P1	P2	P3	P4	P5	P6	P7	P8	P9
First symptoms	J	J	J	J	J	J	J	J	J
Age at first symptoms (d)	4	2	1	7	2	28	5	3	4
Other symptoms and signs									
Acholic stools	+	+	+	-	+	+	+	-	-
Hepatomegaly	+	+	+	+	+	+	+	+	+
Splenomegaly	+	+	+	+	+	+	+	+	+
Liver function tests (LFTs)									
Age at tests (d)	35	33	44	62	89	53	79	97	60
TB (μmol/L)	164	168	211	141	125	287	56	158	104
DB (μmol/L)	136	102	119	108	104	229	46	117	58
ALT (U/L)	55	59	41	110	152	117	63	72	58
AST (U/L)	206	199	309	338	371	250	174	283	177
GGT (U/L)	144	254	54	147	222	126	49	259	167
TBA (μmol/L)	69	96	96	84	66	114	59	158	106
Alb (g/L)	34.6	35.0	38.4	41.8	32.2	40.2	36.3	39.7	43.3
Glu (mmol/L)	4.0	ND	6.0	4.2	2.9	2.5	4.1	2.0	1.9

P, patient; J, jaundice; ND, not done; TB, total bilirubin; DB, direct bilirubin; ALT, alanine aminotransferase; AST, aspartate aminotransferase; GGT, γ-glutamyl transpeptidase; TBA, total bile acid; Alb, albumin; Glu, glucose.

-, negative; +, positive.

**FIGURE 1:**
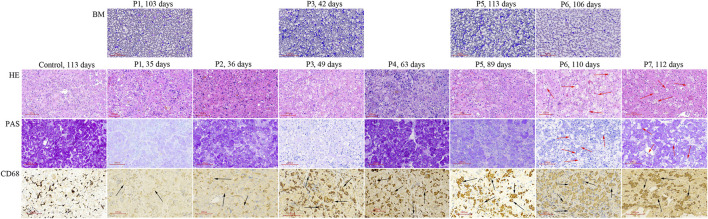
Histologic studies of bone marrow aspirations and liver tissues obtained from NP-C patients presenting as neonatal cholestasis. Vacuolated Kupffer cells (black arrow) are observed in CD68 staining sections from liver tissues of all 7 NP-C infants (P1 ∼ P7), but not in control (a neonatal cholestatic infant with unknown cause). Foam cells (red arrow) are observed in HE and PAS sections of P6 and P7 when vacuolated Kupffer cells with enlarged sizes become obvious. BM, bone marrow aspiration; LS, liver specimens; P, patient.

P1 ∼ P7 underwent liver biopsy. Foam cells were only detected in HE staining sections of P6 and P7, but vacuolated Kupffer cells were detected in CD68 staining sections from all 7 liver tissues (Figure 1) Vacuolated Kupffer cells were scattered in P1 and P2, whose liver tissues were obtained at the age of 35 and 36 d, respectively. More vacuolated Kupffer cells were identified in P3 and P4 at the age of 49 and 63 d, respectively. Most Kupffer cells detected lipid vacuoles in P5 at 89 d of age, and a few had enlarged sizes. Vacuolated Kupffer cells with enlarged sizes became obvious in P6 and P7 at the age of 110 and 112 d, respectively.

Bone marrow aspiration was performed in P1, P3, P5, and P6 at ages ranging from 42 to 113 d, but no foam cell was observed (Figure 1).

### Significance of Vacuolated Kupffer Cells

Of the 168 enrolled neonatal cholestatic infants, 26 detected vacuolated Kupffer cells, including 19 with splenomegaly (Table 3). Six of the 26 infants (P1 ∼ P5, and P7) were diagnosed as NP-C, comparing to none of the 142 infants without vacuolated Kupffer cells (6/26 vs. 0/142, *χ* ^*2*^ = 33.983, *p* < 0.001). The ratio of positive diagnosis of NP-C was 23.1% (6/26) in neonatal cholestatic infants with vacuolated Kupffer cells, and it could increase to 31.6% (6/19) in neonatal cholestatic infants with both vacuolated Kupffer cells and splenomegaly.

**TABLE 3:** Basic information of the 168 neonatal cholestatic infants with unexplained causes.

	With vacuolated Kupffer cells (*n* = 26)	Without vacuolated Kupffer cells (*n* = 142)
Gender (male/female)	14/12	92/50
Hepatomegaly	26 (100%)	142 (100%)
Splenomegaly	19 (73.1%)	47 (33.1%) ^*^
Age at liver biopsy (d)	68 [46, 90]	73 [59, 100]
NP-C (*NPC1*)	6 (23.1%)	0 (0.0%) ^*^
NP-C (*NPC2*)	0	0

NP-C, Niemann-Pick disease type C.

Interquartile range in square brackets.

## Discussion

The diagnosis of NP-C is often delayed in neonatal cholestasis. The measurement of biochemical markers, such as plasm oxysterols, is recommended for early detection of NP-C (Vanier et al., 2016; Geberhiwot et al., 2018), while liver biopsy is now rarely needed (Geberhiwot et al., 2018; Patterson, 2000). Disappointingly, oxysterol screening has a relatively low specificity on distinguishing NP-C from other causes in neonatal cholestasis (Polo et al., 2016; Degtyareva et al., 2019). The NP-C infants with neonatal cholestasis usually still have liver biopsy done for etiologic studies because clinical manifestations are non-specific in the early disease course (Yerushalmi et al., 2002; Evans et al., 2017). Liver foam cells, a typical light microscopic feature of NP-C, can raise a high clinical suspicion of NP-C, but are detectable in only 37–50% NP-C children (Kelly et al., 1993; Rodrigues et al., 2006). In this study, liver foam cells were detected in 2 (28.6%) NP-C infants beyond 3 months of age.

Differentiated from liver foam cells, vacuolated Kupffer cells were detected in all 7 NP-C infants who underwent liver biopsy at age ranging from 35 to 112 d. Abundant vacuolated Kupffer cells were detected at age 89 d and a few had enlarged sizes. When vacuolated Kupffer cells with enlarged sizes became obvious, liver foam cells were observed. A previous study also found that liver foam cells were negative in the early disease course and developed with age (Yerushalmi et al., 2002). These indicate that vacuolated Kupffer cells can evolve into liver foam cells. NP-C was finally diagnosed in 23.1% of neonatal cholestatic infants with vacuolated Kupffer cells, but none of those without vacuolated Kupffer cells. Hence, the presence of vacuolated Kupffer cells raises a clinical suspicion of NP-C in neonatal cholestatic infants, especially in those with splenomegaly, while their absence excludes a possibility of NP-C.

Demonstration of foam cells in bone marrow adds to clinical suspicion of NP-C (Kelly et al., 1993; Geberhiwot et al., 2018), but it can be negative in early infancy (Rodrigues et al., 2006). It is believed that foam cells may become apparent in bone marrow as the disease evolves. In the current study, foam cells were not identified in all 4 bone marrow samples obtained within 4 months of age. Therefore, the diagnosis of NP-C may be missed if early bone marrow aspiration is only relied on. It challenges the importance of bone marrow aspiration for the diagnosis of NP-C in younger infants with neonatal cholestasis.

NP-C infants usually present as neonatal cholestasis with high GGT (Woś et al., 2016; Yamada et al., 2019), but in some instances as neonatal cholestasis with low GGT (Evans et al., 2017). In the current study, we found 2 NP-C infants presented as neonatal cholestasis with low GGT (<100U/L). Furthermore, 4 NP-C infants were found to have fasting hypoglycemia. The reasons of hypoglycemia are still unclear. It may be associated with mitochondrial dysfunction which has been demonstrated in fibroblasts derived from NP-C patients (Woś et al., 2016). Therefore, blood glucose should be routinely monitored in NP-C infants presenting as neonatal cholestasis.

## Conclusion

This study reports the molecular and clinical findings of 9 neonatal cholestatic infants diagnosed as NP-C. Vacuolated Kupffer cells are detected in all 7 NP-C infants who underwent liver biopsy in early disease course. The presence of vacuolated Kupffer cells raises a clinical suspicion of NP-C in neonatal cholestatic infants, especially in those with splenomegaly.

## Data Availability

The datasets for this article are not publicly available due to concerns regarding participant/patient anonymity. Requests to access the datasets should be directed to the corresponding author.
